# Being in Virtual Reality and Its Influence on Brain Health—An Overview of Benefits, Limitations and Prospects

**DOI:** 10.3390/brainsci14010072

**Published:** 2024-01-10

**Authors:** Beata Sokołowska

**Affiliations:** Bioinformatics Laboratory, Mossakowski Medical Research Institute, Polish Academy of Sciences, 02-106 Warsaw, Poland; beta.sokolowska@imdik.pan.pl

**Keywords:** perception, cognitive and motor imagery, brain health/disorders, virtual reality, novel diagnosis and treatment

## Abstract

Background: Dynamic technological development and its enormous impact on modern societies are posing new challenges for 21st-century neuroscience. A special place is occupied by technologies based on virtual reality (VR). VR tools have already played a significant role in both basic and clinical neuroscience due to their high accuracy, sensitivity and specificity and, above all, high ecological value. Objective: Being in a digital world affects the functioning of the body as a whole and its individual systems. The data obtained so far, both from experimental and modeling studies, as well as (clinical) observations, indicate their great and promising potential, but apart from the benefits, there are also losses and negative consequences for users. Methods: This review was conducted according to the PRISMA (Preferred Reporting Items for Systematic Reviews and Meta-Analyses) framework across electronic databases (such as Web of Science Core Collection; PubMed; and Scopus, Taylor & Francis Online and Wiley Online Library) to identify beneficial effects and applications, as well as adverse impacts, especially on brain health in human neuroscience. Results: More than half of these articles were published within the last five years and represent state-of-the-art approaches and results (e.g., 54.7% in Web of Sciences and 63.4% in PubMed), with review papers accounting for approximately 16%. The results show that in addition to proposed novel devices and systems, various methods or procedures for testing, validation and standardization are presented (about 1% of articles). Also included are virtual developers and experts, (bio)(neuro)informatics specialists, neuroscientists and medical professionals. Conclusions: VR environments allow for expanding the field of research on perception and cognitive and motor imagery, both in healthy and patient populations. In this context, research on neuroplasticity phenomena, including mirror neuron networks and the effects of applied virtual (mirror) tasks and training, is of interest in virtual prevention and neurogeriatrics, especially in neurotherapy and neurorehabilitation in basic/clinical and digital neuroscience.

## 1. Introduction

### 1.1. Basic Features of Virtual Environments

The essence of VR is the experience of being in computer-generated interactive worlds. This makes it possible to evoke physiological and psychological reactions similar to real ones [1,2,3]. In addition, it is possible to control the virtual environment (VE) to eliminate many influencing and interfering factors, giving the VE a high ecological value [1,2,3,4,5].

Virtual reality is described by three basic features: immersion, sense of presence and interaction. Immersion (an objective feature) is the sensual context of the experienced reality providing sensory stimuli that give the impression of being in the digital reality. Immersion is primarily affected by the quality of the equipment used. The more high-quality sensory stimuli the system provides, the better its fidelity to the real world. With infinitely high immersion, our brain would not see the difference between the real world and the computer-created one. The second feature of VR is the sense of presence (a subjective feature), i.e., the psychological perception of being involved in (or being part of) VR. People in VEs react realistically, while the degree of realness is determined by the experienced illusion of the place and its probability. Reactions range from physiological arousal to emotional and behavioral responses of participants in virtual worlds. This emphasizes that the important aspect of this presence is participant engagement in VR. The third feature of VR is interaction, which is related to the computer’s ability to detect the subject’s actions and respond to them in real time.

**Figure 1:**
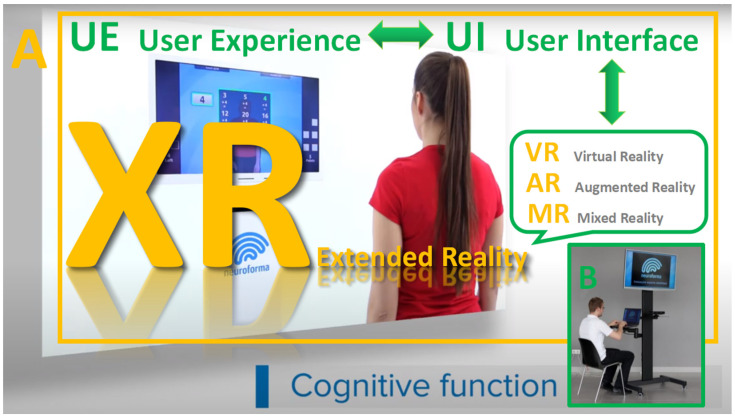
Illustration of XR and its classical components VR, AR and MR, although their areas can overlap. Panel (**A**) presents an example of a non-immersive virtual system, where the participant stands in front of the screen and sees herself surrounded by virtual objects (user interface, UI) performing the most accurate test task (user experience, UE). Using the biofeedback and prompts of the system assistant, as well as the precise preparation of the training sessions according to the activity/test goal by the operator–researcher in panel (**B**), the participant is emotionally engaged in the best possible execution of the virtual task (from our lab repository, with the permission of Titanis Ltd., Warsaw, Poland).

Nowadays, advanced and attractive extended reality (XR) refers to novel technologies such as virtual reality (VR immerses users in a computer-generated environment), augmented reality (AR superimposes digital information onto a user’s view of the real world) and mixed reality (MR mixes VR and AR by combining elements of virtual and real environments) as shown in Figure 1 [2,6,7]. XR environments and tools play significant roles in both basic and clinical neuroscience as well as in modern medical practice due to their high accuracy, sensitivity and specificity and, most importantly, their high ecological value [4,5,8,9,10].

### 1.2. Development of Virtual Environments

**Figure 2:**
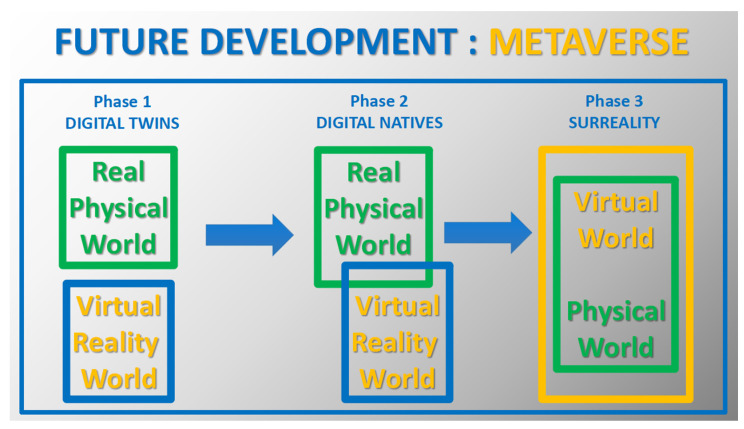
Illustration of the creation of the concept of digital development as the Metaverse: the first phase as digital twins (real physical and virtual reality worlds), the second phase as extended reality and the future perspective (the third phase) as the Metaverse environment.

**Figure 3:**
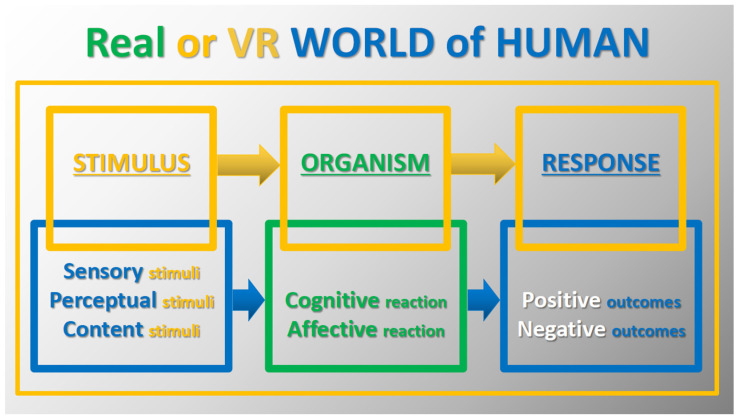
Illustration of the stimulus–organism–response (SOR) concept with the human world may be presented in the following steps: exposure to various stimuli and the body’s response to them, which ultimately leads to positive or negative outcomes.

Today, XR development offers an innovative concept for the digital future of the current world, dubbed the “Metaverse”, aimed at enhancing virtual experiences and creating a digital world that is complex, interactive and connected to the real world [11,12]. This would be a digital environment for communicating, shopping, educating, working and doing everything that would normally be planned and executed online. Although there are already projects related to this, many neuroscientists, other researchers and experts point to emerging limitations and possible/potential risks [13,14,15]. Figure 2 illustrates this new concept within the framework of the observed subsequent stages of development of information technology (IT) and information and communication technology (ICT) [15,16]. The general concept of modeling research in the real and virtual human world can be presented in the following steps: exposure to various stimuli and the body’s reaction to them, which ultimately leads to positive or negative effects, as shown in Figure 3 [17,18].

### 1.3. Research Area, Objectives and Research Hypothesis/Premise

The research covered in this review is primarily human neuroscience, with an emphasis on brain research. The review is based on the basic premise that the current extremely rapid technological development not only has observed and documented benefits, but can also be a source of potential risks. Some of the negative effects associated with being in VEs are presented. Directions for development and steps being taken to eliminate them are also indicated. The prospects for future generations to live in a completely new, integrated digital environment, covering the basic areas of their activity, are outlined.

## 2. Methods

### 2.1. Identification and Selection of Articles

**Figure 4:**
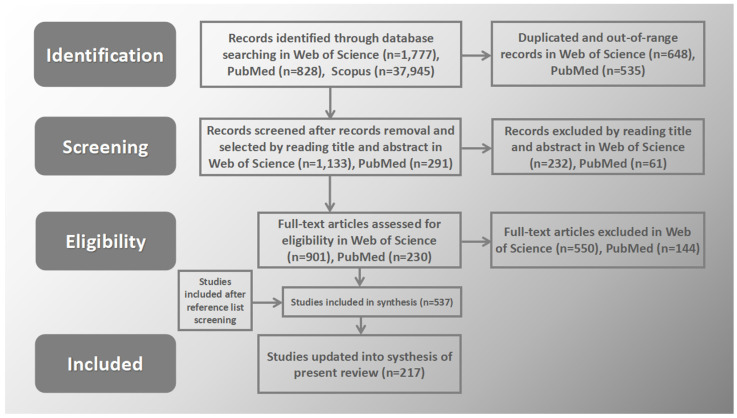
Illustration of article identification and screening process flow diagram (adapted from PRISMA, i.e., Preferred Reporting Items for Systematic Reviews and Meta-Analyses).

The electronic databases Web of Science Core Collection; PubMed; and Scopus, Taylor & Francis Online and Wiley Online Library (the latter to complete the obtained core datasets) were searched, and the focus was primarily on the post-COVID-19 period. This is due to the fact that most of the selected articles (more than half of them) were published in the last five years and represent state-of-the-art approaches and new results/discoveries. The pandemic and lockdown were a period of extraordinary acceleration in the development of innovative IT/ITC technologies and the resulting widespread use of these technologies both in everyday life and in medical and scientific research centers or institutes. The literature search was conducted in accordance with the PRISMA (Preferred Reporting Items of Systematic Reviews and Meta-Analyses) guidelines as depicted in Figure 4 [19].

### 2.2. Eligibility Criteria

The analysis included English-language articles that (1) used virtual environments that were not only immersive, but also non-immersive or semi-immersive, and (2) addressed key application areas in neuroscience and medicine, including brain health. The author chose to focus on the term “virtual reality”, which also included interesting novel combined/mixed environments (such as “augmented reality”, “mixed reality”, “extended reality”). If both conditions were not met, the paper was excluded. Final inclusion decisions were made by consensus, and articles were grouped based on topics in basic and clinical neuroscience, medical practice (current and future) and data type. The selected articles were analyzed for information on the virtual technology model used, proposed and applied programs/sessions and procedures, defined and/or testing/validating conditions/groups (e.g., traditional vs. virtual), number of subjects, and documented favorable and unfavorable/adverse results of being in virtual worlds.

## 3. Results and Discussion

### 3.1. VR Approaches as Novel Beneficial Environments/Tools and Discussion on Their Significance in Neuroscience

#### 3.1.1. Traditional Versus Virtual Research Approaches

In neuroscience, neuropsychology plays a key role in brain and behavior research using VR, and methods for verifying the effects in VEs are usually classic neuropsychological tools [20,21,22,23]. This leads to interesting comparisons between conventional and innovative tools, often in favor of virtual ones. The ecological limitations of traditional neuropsychological testing and some difficulties in conducting tests or training in real-life scenarios have paved the way for the use of VR-based tools. VR tests are often based on “real-world” tasks such as behavior in the classroom, kitchen, supermarket or street [21,22,23]. Therefore, most of these tests are designed to assess executive functions (EFs) and the interactions between various cognitive and sensorimotor processes using real-life task patterns. Moreover, the engaging form of VR testing is an interesting alternative to classical neuropsychological tests that require a high level of attention [20].

**Figure 5:**
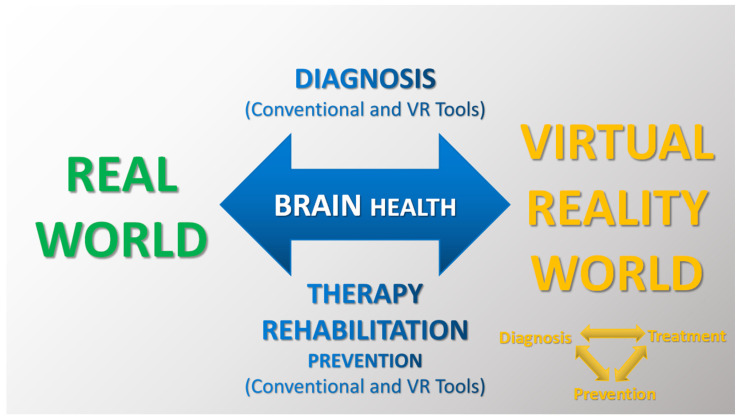
Illustration of basic brain research tools in both real (conventional approach) and virtual (VR approach) environments for modern diagnosis, therapy, rehabilitation and prevention.

**Figure 6:**
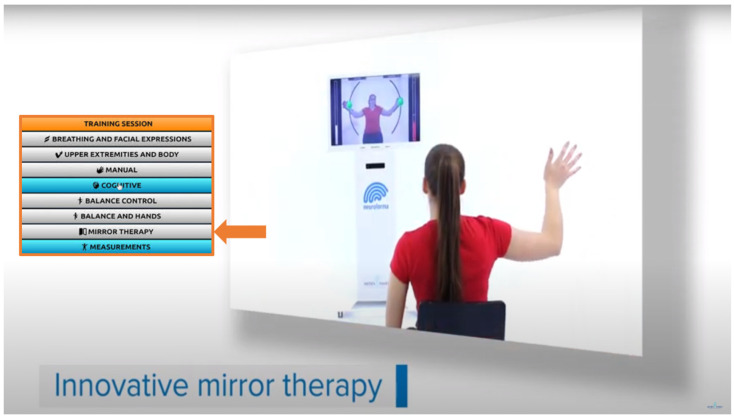
Illustration of a VR training session with a mirror therapy option: the participant exercises one hand, but sees an image of herself with both hands exercising in a non-immersive VR system (which is at MMRI PAS, from our lab repository, with Titanis’s permission).

Scientists indicate that VR has the potential to become the gold standard in neuropsychological diagnostics. Innovative VR technologies are computer–user interface platforms that implement real-time simulation of an action or environment, enabling participant interaction via multiple sensory modalities. As a result, VR diagnosis can be very effective, and similarly, VR treatment can be an effective intervention and support for improving multiple functions and skills in participants’ virtual worlds [24,25]. Figure 5 and Figure 6 illustrate that the VR tools can be used both to diagnose and treat dysfunctions and deficits of body systems/organs and to provide an environment for adaptation to daily life after treatment, as well as for prevention and support of natural aging processes [20,21,22,23,24,25].

#### 3.1.2. Basic Benefits of Using Virtual Environments

It can be noted that in addition to the remarkable diagnostic value of VEs, a number of findings demonstrate that VR training/exercise can have a positive impact on an individual’s (neuro)physiological, (neuro)psychological and (neuro)rehabilitation outcomes compared to traditional training and exercise [26,27]. Neuroscientists point out that classical neuropsychological tests/tasks have certain limitations in terms of generalizing their results, while the results obtained in VEs can be extrapolated to real (actual) functioning due to the high ecological validity of VEs (while maintaining the laboratory precision of the measurements) [21,22,23,24,25,26,27]. It is indicated that the advantage of VR is a higher degree of objectivity compared to clinical interviews or self-report methods, which are largely dependent on the circumstances, including unreliable memory (as a result, VR can effectively support and even verify classical approaches). Also of interest are researchers’ observations that VR seems to allow for a more realistic simulation of social interactions compared to standard methods of testing personal space, such as the use of photographs or abstract verbal stimuli, as well as traditional methods of assessing emotions based on role-play tests, in which the effect depends on the individual’s imagination and the examining person. Whether the improvement observed in the VE can be generalized to patients’ daily functioning remains an open question. Nevertheless, a number of studies point to this possibility (Table 1). In addition, every participant in the digital world knows that everything depicted in it is not real. At the same time, the mind and body behave as if it were real after all. This makes it easier for people to face difficult situations or test new therapeutic strategies. A feature of exposure therapies in VEs is the therapist’s ability to constantly adjust the parameters of the environment to match the patient’s actions and feelings. This allows the therapist/system to tailor the level of difficulty to the specific patient, thus providing a highly personalized therapeutic program. The Neuroforma environment, which we use in our modeling studies, works in a similar way [28,29,30].

#### 3.1.3. Examples of Research Area on the Impact of Virtual Environments on (Brain) Health

Digital reality is constantly evolving, so its impact on human health is changing and requires the updating of knowledge. On the other hand, there are already many areas of the use of virtual technologies, such as precise (neuro)diagnostics and effective support in the treatment of a wide range of diseases (also those related to the nervous system), including the latest findings in neuroscience, such as the phenomena of (neuro)plasticity or mirror neuron networks [31,32,33].

For example, the Riva, Cavedoni and Kourtesis teams conducted neuroscientific research to propose, develop, test and validate various models of VR technology (e.g., different levels of immersion) for healthy and patient populations [2,9,20,34,35]. Their novel studies and others [5,21,22,23,24,25] illustrate the benefits of using VR and demonstrate new findings on brain structure/function and plasticity. In addition, Bonini and co-workers [36] provide an interesting summary of 30 years of research on mirror neurons (MNs) from the first description by Rizzolatti’s group [37,38,39] as a class of monkey premotor cells discharging during both action execution and observation to current implications and applications in humans. A recent study by Thompson’s team [40] demonstrates that mirror neuron brain areas contribute to action identification, but not intention. Zhou and colleagues [41] suggest that the configuration of an action observation network depends on the observer’s goals. Plata-Bello’s group [42] analyzed patterns of brain activity during the observation of painful expressions and assessed the relationship between this activity and interpersonal reactivity index (IRI) scores. For non-invasive brain stimulation, authors concluded that observing painful expressions triggers activation in sensorimotor MNs, and this activation is influenced by a person’s level of empathy. Studies of the MN system and neural plasticity using VR environments [43] involving data from electroencephalography, neuroimaging and non-invasive brain stimulation [44,45,46,47] present innovative multidisciplinary treatment models based on the mixed methodologies and/or objective (neuro)physiological signals. Recent findings demonstrate novel individualized biomarker-based approaches with a well-targeted patient population in neurotherapy and neurorehabilitation, for example, individuals with schizophrenia and autism spectrum disorders and individuals with neurological or neuromuscular diseases [48,49,50,51,52,53]. Table 1 presents examples of VR use, as well as comparisons of traditional methods with new digital proposals. Additionally, research using VEs can provide recommendations for their specific application, as well as aiding in the validation and standardization of VEs.

**Table 1:** Examples of areas of use of digital environments with the participation of healthy individuals (experimental and modeling studies) and various patient populations (proposed diagnostic, therapeutic and preventive approaches) in basic and clinical neuroscience. Today we can observe not only the rapid development of innovative technologies but also their implementation in many different areas of modern human activity. In the future, digital environments may constitute the basis for the functioning of human societies.

Applications of VEs	Authors	Descriptions of VR Approaches, Basic Results and Conclusions
Experimental and modeling studies with healthy participants in real and virtual environments (VEs)	Kodithuwakku et al. 2024 [4]	Researchers investigated the effects of virtual heights, dual-tasking (DT) and training on static postural stability in healthy adults without simulator/motion sickness and acrophobia. The results of modeling VEs showed that static balance deteriorates at higher VR altitudes and during DT and improves with VR training (but excessive visual stimulation reduced cortical response and postural control ability [33]). The authors suggest that VR can be used as a potential tool for ergonomic balance training (e.g., as strategies to prevent falls in workplaces).
	Benelli et al. 2023 [54]	Researchers emphasize that there is not yet an effective solution to the problem of cybersickness (CS) and propose an innovative approach for a frequency-dependent reduction in CS in VR via transcranial oscillatory stimulation of the vestibular cortex. The authors indicate that the new approach may be used to treat a variety of vestibular dysfunctions.
	Brock et al. 2023 [55]	The modeling study examined movement kinematic and postural control for visual–motor skills during golf playing in real and VR environments in novice golfers (students). The results showed differences in putter swing between real and virtual reality, as well as between VE with and without haptic information. The authors note the possibility of different motor learning transfers in the conditions tested.
	Sokołowska 2021 [28,29]	The research proposed a novel model-based approach to assessing functional lateralization of the brain and demonstrated highly effective recognition of functional and postural asymmetries using non-immersive VE in healthy adults.
Pain Procedural pain Neuropathic pain Phantom limb pain (PLP)	Li et al. 2023 [56]	The research team proposed an interesting project to explore the analgesic effect of VE in healthy adults. The researchers compared the effect of immersive VR and no VR control on pain perception. The authors pointed to the analgesic benefits of VR and concluded that the VR findings support further development of digital healthcare.
	Phelana et al. 2023 [57]	The study describes the process of designing, testing and implementing a VR system in a hospital setting. In the experimental phase, the study was conducted on healthy adults, and pain was induced through cold pressor. The effectiveness of the VR system was then tested on burn-injured patients. The results show that prolonging the use of VR after a therapy session can help treat procedural pain more effectively.
	Aurucci et al. 2023 [45]	Researchers proposed novel non-pharmacological interventions, such as transcutaneous electrical nerve stimulation (TENS) to activate peripheral pain relief via neuromodulation and VR to modulate patients’ attention. This is an example of a brain–computer interface enabling personalized multisensory intervention in neuropathic pain (i.e., a comprehensive approach to individualized therapy). The study demonstrates the feasibility of real-time pain detection based on objective neurophysiological signals and the effectiveness of a triggered combination of VR and TENS to significantly reduce neuropathic pain.
	Annapureddy et al. 2023 [58]	Scientists tested a mixed reality system for treating phantom pain, using the immersive Mr. MAPP environment with a novel in-home virtual mirror therapy option. Mirror therapy allows participants to visually see missing limbs using a mirror. The results show that VE can potentially relieve pain and improve function in PLP patients.
	Hali et al. 2023 [31]	Based on the current literature, the authors document that VR therapy has the potential to effectively treat PLP, and they identify additional benefits by adding vibrotactile stimuli to VR therapy. This approach leads to even greater pain reduction compared to VR therapy alone.
Acquired brain injury (ABI) Traumatic brain injury (TBI) Intensive care unit weakness (ICU-AW)	Bulle-Smid et al. 2023 [59]	Researchers provide a review of extended reality (XR) environments as particularly promising in rehabilitating people with ABI and promoting professional supervision, faster recovery, shorter hospital stays and lower expenses. The authors suggest that future XR research should focus on developing appropriate XR environments, improving the safety and support for both patients and healthcare professionals.
	Calabro et al. 2023 [60]	The results of a study using non-immersive VE in tele-neurorehabilitation of patients with severe ABI (sABI) demonstrate that the VR approach is a suitable alternative and/or complementary tool to improve motor and cognitive function and reduce behavioral changes in sABI patients. In addition, the authors indicate a beneficial effect on alleviating caregivers’ distress and promoting positive aspects of caregiving.
	Brassel et al. 2021 [26]	Researchers indicate that VR is increasingly being used to assess and treat impairment resulting from ABI due to its perceived advantages over conventional methods. In addition, the authors emphasize that there are no tailored options for designing and implementing VR in ABI or TBI rehabilitation. The researchers made some recommendations regarding these issues in this patient population.
	Keller et al. 2020 [5]	Researchers presented that VR-based therapy to regain upper extremity function induces changes in the cortex grey matter in persons with ABI. The researchers proposed an interesting interactivity VR game in which ABI patients with upper limb paresis use an unaffected limb to control a standard input device and a regular computer mouse to control virtual limb movements and tasks in a virtual world. The results showed that the VR rehabilitation program significantly improved motor functions and skills in the affected upper extremities of subjects with ABI. In addition, significant increases in grey matter volume in the motor and premotor regions of the affected hemisphere and correlations of motor skills and volume in non-affected brain regions were observed, pointing out marked changes in structural brain plasticity.
	Castelli et al. 2023 [61]	This is an interesting paper on the role of technology-based rehabilitation in patients with intensive care unit weakness (ICU-AW). The results of the study show that intensive structured rehabilitation is effective in improving motor function, disability and quality of life of patients with severe acquired brain injury and acquired weakness. For example, a combination of non-immersive VR training and focal muscle vibration can result in significant improvements in overall disability and quality of life compared to traditional treatment alone. The researchers recommend VEs in the neurorehabilitation of ICU-AW patients to facilitate the fastest possible neurorepair.
Multiple sclerosis (MS)	Milewska-Jędrzejczak and Głąbiński 2023 [32]	The research group presents recent findings of brain plasticity induction and its beneficial impact after both traditional physical and VR-based rehabilitation in patients with multiple sclerosis. The basic premise of this approach is that physical rehabilitation and physical activity are known non-pharmacological treatments for MS.
	Kamm et al. 2023 [62]	The study presents a new home-based immersive dexterity training program for MS patients based on a VR headset. The study demonstrates good feasibility, usability and patient engagement and satisfaction with this VR training (VRT) program. The results also indicate an improvement in the motor skills of the dominant hand after VRT.
	Cortés-Pérez et al. 2021 [63]	The authors analyze and demonstrate that VR-based therapies are effective in reducing fatigue and have a positive impact on patients’ quality of life.
	Leonardi et al. 2021 [64]	This clinical study on VR-based neurorehabilitation of cognitive dysfunction in people with relapsing/remitting MS showed improvement in mood and visuospatial skills. The researchers suggest that VR can be a motivating and effective tool for cognitive recovery in persons with MS.
Stroke	Bedendo et al. 2024 [65]	Researchers emphasize that to prevent deterioration of mobility, patients undergoing chronic rehabilitation must perform well-focused and repetitive exercises. In their view, VR appears as an interesting tool that offers the possibility of training and measuring patient performance. The authors proposed and tested an exercise design for the recovery of stroke patients at home, considering standard measures related to usability, immersion, workload and adverse symptoms, and with the involvement of rehabilitation experts. The results suggest the promising potential of VR applications for the future development of home rehabilitation programs.
	Bargeri et al. 2023 [66]	Researchers investigate and compare the efficacy and safety of VR rehabilitation for motor upper limb function and activity after stroke in immersive, semi-immersive and non-immersive modes of VR intervention with or without traditional therapy versus conventional therapy alone. The authors recommend the feasibility of using VR technology in clinical practice.
	Cinnera et al. 2023 [67]	The authors explored the use of immersive VR to treat visual perception in unilateral spatial neglect (USN) after a stroke. The results demonstrate not only the potential benefits of VR in treating visual perception impairment in USN, but also that VE motivates patients during the rehabilitation process, improving compliance and interest.
	Errante et al. 2022 [68]	Researchers investigated the effectiveness of a new VR rehabilitative approach with action observation therapy (AOT) based on the discovery of mirror neurons to improve motor function. The study evaluated action observation (AO) added to standard VR (AO + VR) to improve upper limb function in stroke patients, compared to a control treatment consisting of observing naturalistic scenes (CO) without any action content, followed by VR training (CO + VR). The authors suggest that AO + VR therapy could be adjunct to currently available rehabilitation interventions for post-stroke recovery and could be used as part of standard sensorimotor training or in individualized (tele)rehabilitation.
	Wiley et al. 2022 [25]	The review examined the effects of VR therapy on cognition after stroke. The authors indicate that VR therapy (a) is a promising new form of technology that improves patient satisfaction with post-stroke rehabilitation; (b) has the added advantages of providing immediate feedback and a degree of difficulty that can be easily modified (i.e., the user-friendliness of this form of rehabilitation); and (c) has the potential to improve various motor, cognitive and physical deficits after stroke. In summary, VEs can be useful in rehabilitation settings.
Mild cognitive impairment (MCI)	Gómez-Cáceres et al. 2023 [69]	Researchers analyzed and evaluated the effectiveness of VR-based neuropsychological interventions in improving cognitive functioning in patients with MCI. The authors showed that VEs have a beneficial effect on improving cognitive functioning in patients with MCI, providing a basis for clinical practice recommendations.
	Yang et al. 2022 [70]	The results of the study show that VR-based cognitive training and exercise training improve brain health and cognitive and physical function in older adults with MCI.
	Liao et al. 2020 [71]	The research team presented the potential of VR-based physical and cognitive training designed as an intervention for cognition and brain activation in elderly patients with MCI. The authors also analyzed whether a VR program designed around functional tasks can improve the instrumental activities of daily living (IADL) of these patients. The results showed that in both training programs without and with VR, there were improvements in executive function and verbal memory (immediate recall). But only in VR were there significant improvements in global cognition, verbal memory and IADL. In the authors’ opinion, VR training can be implemented for older adults with MCI.
	Mancuso et al. 2020 [72]	The authors present the greater benefits/better effects of using VR with non-invasive brain stimulation, VR-NIBS, in the cognitive rehabilitation of patients with MCI and Alzheimer’s dementia.
	Cassani et al. 2020 [47]	Based on the current literature, the authors demonstrated the benefits of the VR-NIBS combination for five therapeutic applications, namely neuropathic pain, cerebral palsy, stroke, multiple sclerosis, and post-traumatic stress disorder (PTSD) and phobias.
VR exposure therapy (VRET) for posttraumatic stress disorders (PTSDs) and specific phobias	Siehl et al. 2023 [73]	The study found that PTSD patients differ in brain activation from control subjects in regions such as the hippocampus, amygdala and ventromedial prefrontal cortex in processing unpredictable and predictable contexts. The researchers suggest that (a) deficient encoding of more complex configurations may lead to a preponderance of cue-based predictions in PTSD and (b) exposure-based therapies need to focus on improving the predictability of contextual processing and reducing enhanced cue reactivity.
	Binder et al. 2022 [74]	Researchers developed a fully automated experimental procedure using immersive VR involving behavioral search, forced-choice and an approaching task with varying degrees of freedom and stimulus relevance. In this study, scientists examined the sensitivity and feasibility of these tasks to assess avoidance behavior in patients with specific phobias. The results show the beneficial effects of immersive VR on specific phobias. In addition, the authors conclude that the behavioral tasks are well suited for assessing avoidance behavior in participants with phobias and provide detailed insights into the avoidance process.
	Alvarez-Perez et al. 2021 [75]	Researchers emphasize that cognitive-behavioral therapy (CBT) with exposure is the treatment of choice for specific phobias. VR exposure therapy (VRET) has been shown to benefit the treatment and prevention of specific phobias by addressing the therapeutic limitations of exposure to real images. Neuroimaging studies on specific phobias demonstrate various changes in brain activation through CBT with VR exposure.
	Hinze et al. 2021 [76]	The review presents current and future applications of innovative digital technologies in the effective diagnosis and cognitive-behavioral psychotherapy of spider phobia (arachnophobia).
Other areas of application of VEs for Parkinson’s disease (PD), attention-deficit hyperactivity disorder (ADHD), schizophrenia spectrum disorders (SSDs) or autism spectrum disorders (ASDs), depressive disorders	Alavian et al. 2024 [77] Kaplan et et al. 2024 [78] Baugher et al. 2023 [79]	Scientists emphasize that relatively few studies using innovative technologies are conducted in groups of patients with Parkinson’s disease. Not only VEs but also robotic (assistive) technologies are used in rehabilitation and evaluation of its (beneficial) effects. Patients’ family members/guardians are also involved in the research. This is crucial for such patients who require advanced care provided by the patient’s environment. The incidence of Parkinson’s disease, like that of other progressive neurodegenerative diseases, will increase as the world’s population ages. High hopes are being placed on the development of digital environments and tools, such as MR and AI.
	Goharinejad et al. 2022 [80] Coleman et al. 2019 [81]	The use of VR, AR and MR technologies in attention-deficit hyperactivity disorder (ADHD) has increased in recent years. ADHD is a neurodevelopmental disorder characterized by problems with concentration, excessive physical activity and impulsivity. The findings show that innovative technologies are promising tools to improve the diagnosis and management of ADHD.
	Holopainen et al. 2023 [82] Aubin et al. 2018 [83]	Studies to date using VR technology in various symptom domains of schizophrenia spectrum disorders (SSDs) point to completely new possibilities for effective SSD treatment. Researchers indicate that (a) VEs still require more research and validation and (b) there are currently few studies examining the latest technology in the treatment of SSDs.
	Zhang et al. 2022 [84] De Luca 2021 [85]	The number of studies using VR-based technology for individuals with ASD has increased over the last decade. De Luca’s team proposed a novel training program in a VE, documenting improved outcomes and changes in frontoparietal network connectivity after VR rehabilitation training. The current research indicates that (a) VEs are promising and efficacious for the assessment and treatment of ASD and have been found to be acceptable by persons with ASD. Researchers also emphasize that VEs provide fully interactive simulations of real-world settings and social situations that are particularly suitable for cognitive and performance training, including social skills and interaction.
	Yan et al. 2024 [86] Baghaei et al. 2021 [87]	Research indicates that non-pharmacological innovations in (personalized) treatment plans/approaches for depressive disorders also include new VR-based strategies that may be effective in supporting the treatment of patients with depression.

Novel VR-based technologies are constantly developing, and their areas of application are expanding and even overlapping, as we see in neuroscience. Table 1 presents and indicates examples of neuroscientific areas of using virtual environments and tools, including pain management [31,56,57,58], improvement of brain injury patients [5,26,59,60,61], post-stroke [10,20,25,33,65,66,67], prevention, and diagnosis and therapy of many serious illnesses. Examples include diseases such as neurodevelopmental disorders (e.g., attention-deficit hyperactivity disorder, ADHD [22,80,81]); schizophrenia spectrum disorders (e.g., schizophrenia [82,83]); autism spectrum disorders (e.g., autism [84,85]); mood (e.g., depressive disorders [86,87]), anxiety (e.g., panic and phobias [74,75,76]), trauma- and stressor-related (e.g., post-traumatic stress disorder, PTSD [73]), neurocognitive (e.g., Parkinson’s or Alzheimer’s and memory cognitive impairment diseases [23,24,47,69,70,71,72,77,78,79]) and neuromuscular disorders (e.g., multiple sclerosis [32,62,63,64]). In addition, VEs are increasingly being incorporated into research and evaluation of natural aging processes or effective support in (neuro)geriatric care (e.g., preventing falls or improving cognitive function in the elderly) [21,70,71]. Model studies with healthy participants are important and interesting in evaluating/testing new technologies [4,28,29,54,55,56].

Overall, it has been observed that current research approaches primarily (a) compare the effects of traditional methods with those based on VEs; (b) combine traditional and innovative approaches/mixed methodology, e.g., searching for (digital) (neural) biomarkers, additionally taking into account data of EEG, neuroimaging and NIBS, as well as EOG, EMG and other biosignals; (c) present different models of VEs; (d) observe the accompanying beneficial and adverse effects and assess potential risks to eliminate them; and (d) predict the next phases of digital reality development.

Although unusual and unexpected challenges are only beginning to be encountered, VR environments make it possible to expand the scope of research on perception, cognitive and motor imagery, and the effects of different learning and teaching pathways. In this context, studies of neuroplasticity phenomena, including the effects of applied virtual (mirror) tasks and training, are of interest in virtual prevention, neurogeriatrics, neurotherapy and neurorehabilitation [5,31,32,33,36,42,58,61].

### 3.2. Being in VR and Discussing the Impact of Technical Aspects and Adverse Symptoms on (Brain) Health

#### 3.2.1. VR Equipment for Non-Immersion, Partial Immersion and Full Immersion

Virtual environments are offered with different degrees of immersion: non-immersive, partial immersion and full immersion [9,18,23,88,89,90]. We carried out our research in the non-immersive virtual environment created by the Neuroforma system [28,29,30]. Such environments are willingly used due to the fact that there are practically no adverse symptoms related to being in them or participating in virtual tests, tasks and training. Above all, however, VEs with full immersion are very attractive. These environments most often use an HMD (head-mounted display) interface. Nowadays, professional HMD sets, in addition to the classic two small high-resolution screens and a headset, increasingly offer additional equipment such as hand-tracking controllers or gloves for the perfect imitation of hand work, as well as an eye-tracking system, shoes mapping leg movement and a system for tracking the user’s location in space. The amount of information available increases even more when additional equipment allows, for example, the measurement of heart rate or galvanic skin response. Such feedback can be recorded by the system and influence what happens in the virtual environment (which, however, can limit the subject’s freedom in VR). An interesting and unusual development of VR technology is a costume worn over all or part of the body. Every movement of the body is monitored and then mapped to the virtual space, which gives excellent visual–motor synchronicity and is used to create a strong illusion of having a virtual body. Moreover, it is pointed out that the use of first-person perspective in visual–motor synchronization gives an even stronger illusion of virtual body possession. Undoubtedly, the ability to virtually represent a subject’s entire body is one of the most important advantages of the latest VR technology over other types of computer user interfaces. Another interesting VE, although already expensive, is the Cave Automatic Virtual Environment (CAVE), in which images are projected via a projector onto the walls and floor of a small cubic room. In this environment, the participant wears glasses that allow stereoscopic vision, and sound is played through loudspeakers in the room [20]. Furthermore, research suggests that the strength of fully immersive virtual environments will be higher than that of non-immersive VEs, which is supported by findings indicating that higher immersion is associated with a stronger sense of presence, and often with more pronounced emotional reactions. Nevertheless, the relationship between immersion and emotional reactions is not clear, nor are the relationships between specific emotions and the sense of presence in VR yet known.

#### 3.2.2. VR and Adverse Symptoms such as Cybersickness

It has already been mentioned that VR can be a non-immersive environment, as well as low-immersive, semi-immersive and fully immersive. The latter may be responsible for the increased incidence of cybersickness (virtual reality sickness). It is a similar, but not identical, term to the concept of motion sickness or simulator sickness. Cybersickness (CS) most likely results from, e.g., the inconsistency between the sense of movement in the virtual environment and stillness in the real world, according to sensory conflict theory [91]. Its main symptoms include (a) disorientation (systemic and non-systemic dizziness), (b) nausea (belching, unpleasant feeling in the stomach, salivation) and (c) oculomotor symptoms (eye fatigue, difficulty focusing, blurred vision, headaches). These symptoms are exacerbated by various factors, and among the important ones are (a) personal factors: age (the younger the person, the more severe the symptoms), female gender, fatigue, posture (sitting is safest); (b) technical inadequacies (devices/interfaces that are inconvenient to use, image lag and flickering, calibration; and (c) the specifics of the virtual task: a sense of lack of control, too long a virtual session (the longer, the greater the risk of adverse symptoms) [90,91]. It is noteworthy that in simulator sickness, oculomotor complaints predominate, while in cybersickness. it is primarily disorientation [92]. It is estimated that CS symptoms affect 60–70% of HMD users, and their severity is about three times that of simulator sickness. The considerations so far show how serious a problem cybersickness symptoms can be. Hence, intensive research is being conducted to reduce the adverse symptoms associated with being in the digital world [54,90,91,92]. Recommendations are being prepared, and interesting neuroscientific studies are being presented to reduce the risk of adverse symptoms. In addition, various questionnaires are proposed to assess this risk in participants of virtual worlds, for example, by the Stanney [91], Kourtesis [92], Laessoe [93] and Kim [94] groups.

#### 3.2.3. VR and the Development of Validation and Standardization Procedures

Another difficulty worth mentioning is the lack of validation and standardization of VEs, which consequently leads, for example, to difficulties in replicating studies and their results [9,26,69,83]. As a consequence, evaluations of different environments are incomparable and thus less reliable. Furthermore, the very nature of both non-immersive and immersive VR largely depends on the use of vision to navigate and perform virtual tests and tasks. Therefore, the inclusion criteria for participants in many studies include normal or corrected vision [22,23]. Moreover, VR training requires a certain level of cognitive functioning and supports computer interfaces and/or virtual objects [20]. Above all, the final success depends on the motivation of the participants themselves to complete tasks and programs in VEs [25,64,65,67,95]. The difficulties indicated may result in different and/or inconclusive results, especially when, for example, reviewing studies using these modern technologies in different non-clinical and clinical groups. Therefore, when interpreting the results of various VR studies, in addition to methodological diligence, it is necessary to take into account the specifics of the VEs.

#### 3.2.4. Summary

Virtual technologies offer new opportunities and perspectives for physical and/or cognitive exercise to improve human health. An interesting summary of current considerations for the future era of virtual/digital neuroscience is a systematic review by Ali’s team [1]. The authors point out that VR has emerged as an innovative, safe and effective tool for the rehabilitation of many childhood and adult diseases. VR-based therapies have the potential to improve both motor and functional skills across a wide range of age groups through cortical reorganization and activation of various neuronal connections [24,71,73,75]. The great potential of using serious VR-based games that combine perceptual learning and dichoptic stimulation in the rehabilitation of ophthalmic and neurological disorders has been demonstrated. Current research on memory retrieval has been inspired by theories of brain plasticity and discoveries about the nervous system’s ability to reconstruct cellular synapses as a result of interaction with enriched environments [32,76]. Therefore, for example, the use of VR training can play an important role in improving cognitive functions and motor disabilities [25,47,59,62,84,86,95]. VR-based training is currently being researched to prevent and control measurements in ocular diseases such as myopia, amblyopia, presbyopia and age-related macular degeneration [1,96]. As indicated by the dynamic development of IT/ITC (which accelerated even further during the COVID-19 pandemic, including futuristic Metaverse concepts), as well as findings in neuroscience, VR technologies will be more accessible and thus widely used in (digital) healthcare in the future [4,8,12,97]. Finally, it is worth mentioning the important issue of the ethical implications of digital technologies [3,98]. This topic, which represents a new challenge for the future, is already being addressed by many researchers, philosophers and computer scientists, pointing out both benefits and serious dangers (cybersecurity, privacy, lack of general recommendations and methods for validation and standardization of virtual environments and tools) and potential threats (cybersickness, addiction to new technologies, currently unknown negative consequences) to future users of virtual worlds [14,97,99].

### 3.3. Limitations and Future Prospects of Digital Worlds with Artificial Intelligence

#### 3.3.1. Basic Limitations of Virtual Environments

Innovative technologies are very attractive and are developing extremely rapidly. Therefore, we are constantly studying their limitations, including in the context of brain research. An important and perceived serious issue is an addiction to the Internet, and consequently to modern technologies. The problem is so important that extensive research is being conducted on the subject [100,101,102,103,104]. Another serious threat noted among users of virtual worlds is the occurrence of cybersickness. Nevertheless, further development of digital technologies will most likely reduce the appearance of its symptoms. Undoubtedly, an important issue is not only health safety but also the security and regulation of the use of new technologies in general. This certainly includes the protection of privacy, collected VR data and other information about participants of VEs. It should be noted that all of these limitations are very important in the context of IT/ICT technologies becoming cheaper and thus more accessible to large groups of users, both in the healthy population and among patients.

#### 3.3.2. Future Development of VR with Artificial Intelligence

The digital worlds of the future seem even more interesting and fascinating with the new challenges and opportunities associated with the development of artificial intelligence (AI). AI is a broad field that encompasses various disciplines, from computer science, (intelligent) data analysis/retrieval and (bio)statistics, (bio)(neuro)hardware and software engineering, linguistics, medicine and neuroscience to philosophy and psychology [105,106,107]. Another interesting proposed direction for the future development of AI is artificial general intelligence (AGI) [107,108]. AGI would be the ability of a machine to “feel, think and act” like a human. Although AGI does not currently exist, it is already being considered that the next level would be artificial superintelligence (ASI), in which a machine would be able to function in all respects, perhaps even “better” than a human [109]. For now, however, these are only very distant time horizons of the future world in which future human generations will live.

AI is expected to be one of the most important technologies of the future and will therefore have an increasing impact on our lives [110,111,112]. This impact includes not only the collection and processing of data, but especially the fusion of AI with AR/VR technologies, and consequently the emergence of the future Metaverse with AI [113]. Discoveries and knowledge about future new digital worlds and their impact on humans will be a great challenge for brain science [114,115,116,117].

## 4. Conclusions

(1) The purpose of this review was to present interesting findings on the effects of using innovative virtual technologies in neuroscience. Immersion and being in created digital worlds influence the behavior of the brain and body. The significant impact on the human brain is still unknown, especially in the long term.
(2) The data obtained to date, both from experimental and modeling studies and from (clinical) observations, indicate the vast and important potential of digital worlds, but their use can have both beneficial and unfavorable effects, including digital ethical aspects that require further research.
(3) Current VR research on human health (and disease) has shown that digital technologies (a) are attractive and stimulate the rapid development of contemporary civilization and the exploration of human brain capabilities and (b) are promising, motivating, easy to personalize and control, and relatively safe for rebuilding/remodeling motor and cognitive functions in brain health and/or disorders.

From the perspective of the future, it is worth noting that this is only the beginning of research into understanding human brain behavior in the digital world of modern civilization’s development. Today we know for sure that we still know very little not only about ourselves but also about our fundamental and extraordinary organ of cognition of reality—the brain. Moreover, the human brain, functioning in the individual, social and cultural spheres, creates multidimensional realities, which will be true in the Metaverse concept with AI. The possibility of creating the illusion of embodiment opens new research perspectives.

## 5. Limitations of the Present Review

Since general standards and methods for validating VR applications have not yet been developed for innovative IT/ICT technologies, many of the studies presented cannot be compared with each other. In the future, it would be worthwhile to include such a meta-analysis comparing results in different virtual environments, from a non-immersive model through a semi-immersive model to a full immersion. However, there is still no independent virtual standard in scientific research and clinical practice. This is also pointed out by the researchers themselves. Therefore, especially important are those VR studies that also propose validation approaches in similar areas of VR applications. This is also illustrated in the review (Table 1). It is not possible to present the full spectrum of so many proposed and already ongoing VR studies. Even this limited overview shows the many benefits of novel technologies based on virtual environments, while also demonstrating that they may not be neutral to their users (Table 2). The proposed virtual worlds, while very attractive, interesting and increasingly popular, require an extremely cautious approach due to their still-unknown possible negative effects on human health, as well as unregulated privacy/security issues and concerns about digital bioethics.

## Figures and Tables

**Table 2:** Overall benefits, limitations and prospects of VR-based digital worlds.

Benefits of VEs	Adverse Effects and Limits	Other Implications
High ecological value of VEs; users know that everything shown in VR is not real, yet the mind and body behave as if it were real after all.	Addiction to modern technology, acute and/or emotional distress (physical risk, barriers, task/technical difficulties, time pressure, delusions, negative emotions).	Technologies are present in many areas of modern civilization, and VEs have gained popularity in the last decade.
High precision, sensitivity and specificity of VR diagnostics; researchers point to its potential to be the gold standard in neuropsychology.	Limiting contact with the real world and choosing to be in VR more often.	Benefits of VEs outweigh the observed limits, especially in basic/clinical neuroscience and biomedical applications.
Highly effective VR neurotherapy and neurorehabilitation.	Various unfavorable physiological outcomes (e.g., visual and muscular fatigue, musculoskeletal discomfort) and cybersickness.	Intensive research on the elimination of serious limitations is carried out by many groups of scientists, experts, etc.
The effectiveness of VR prevention in everyday life and modern medical practice.	The need to test and validate subsequent generations of VR devices, equipment, environments, etc.	Very dynamic and unpredictable IT/ICT development.
Very attractive, innovative VR models and significant positive motivation and high involvement in VR being.	The most advanced of them are found only in various institutions or centers, while remote and home versions are still in the early stages of their development.	The consequence is the incomparability of the created virtual devices, systems, and environments and their varied impact on users.
Attractive and interactive ways for users to communicate, including biofeedback and access to helpful information and comments.	Lack of general standardization of procedures and use of VR environments in neuroscience.	Inspiration for new directions in neuroscience and novel fields of science, industry, education, sports, military, health service, etc.
Possibility to control and adapt to the current situation, conditions and achieved level of performance of VR tasks or training.	The costs of novel technologies are still high, and there are still no legal regulations maintaining the safety and privacy of users and no general bioethical standards for the use of VR.	Prospects integrate the digital and real worlds using artificial intelligence, taking into account and regulating the mentioned issues regarding users and digital bioethics.

## Data Availability

Not applicable.

## Abbreviations

ABI	acquired brain injury
ADHD	attention-deficit hyperactivity disorder
AGI	artificial general intelligence
AI	artificial inteligence
AO	action observation
AOT	action observation therapy
AR	augmented reality
ASD	autism spectrum disorder
ASI	artificial superintelligence
CAVE	cave automatic virtual environment
CBT	cognitive-behavioral therapy
CS	cybersickness
DT	dual-tasking
EEG	electroencephalogram
EF	executive function
EMG	electromyogram
EOG	electrooculogram
HMD	head-mounted display
IT	information technology
ICT	information and communication technology
ICU-AW	intensive care unit weakness
IRT	interpersonal reactivity index
MCI	mild cognitive impairment
MR	mixed reality
MS	multiple sclerosis
MNs	mirror neurons
NIBS	non-invasive brain stimulation
PD	Parkinson’s disease
PLP	phantom limb pain
PRISMA	Preferred Reporting Items for Systematic Reviews and Meta-Analyses
PTSD	post-traumatic stress disorder
SOR	stimulus–organism–response
SSD	schizophrenia spectrum disorder
TBI	traumatic brain injury
TENS	transcutaneous electrical nerve stimulation
UE	user experience
UI	user interface
USN	unilateral spatial neglect
VE	virtual environment
VR	virtual reality
VRET	VR exposure therapy
VRT	VR training
XR	extended reality
